# Role of rapid antigen detection test for the diagnosis of group-A ß-hemolytic streptococcus in patients with pharyngotonsillitis

**DOI:** 10.1016/S1808-8694(15)30027-6

**Published:** 2015-10-19

**Authors:** Bernardo Cunha Araujo Filho, Rui Imamura, Luiz Ubirajara Sennes, Flávio Akira Sakae

**Affiliations:** a Otolaryngologist (former HCFMUSP resident). ENT Specialist - SBORL – PhD in Otolaryngology -HCFMUSP; b Assistant physician, Otolaryngologist PhD - HCFMUSP; c Associate Professor of Otolaryngology – Medical School – University of São Paulo; d Otolaryngologist (former HCFMUSP resident). ENT Specialist - SBORL – PhD in Otolaryngology -HCFMUSP Otolaryngology Department – University Hospital – Medical School of the University of São Paulo.

**Keywords:** rapid antigen detection test, group A ß-hemolytic streptococcus, pharyngotonsillitis

## Abstract

Group A ß-hemolytic streptococcus (GAS) is an important pharyngotonsillitis etiologic agent. Correct etiologic diagnosis and early treatment prevent suppurative and non-suppurative complications of streptococcal pharyngotonsillitis, however, clinical diagnostic methods are not reliable. Within this context, rapid detection methods of GAS antigen are useful to diagnose this agent.

**Aim:**

The objective of the present study was to determine the sensitivity and specificity of rapid GAS antigen detection tests used in Brazil.

**Study Design:**

Clinical prospective.

**Methods:**

Eighty-one patients with clinical diagnosis of acute pharyngotonsillitis seen at the otorhinolaryngology emergency department of University Hospital, FMUSP, between May 2001 and April 2002, were submitted to two simultaneous collections of oropharyngeal material using swabs. Rapid GAS antigen detection test was compared to culture on blood agar, the gold standard for the diagnosis of this etiologic agent.

**Results:**

Among the 81 studied patients, the rapid test was positive in 56% and negative in 44%. GAS growth in culture was observed for 40.7% of the patients. The sensitivity and specificity of the rapid test were, respectively, 93.9% and 68.7%, and the negative and positive predictive values were 94.2 and 67.4%, respectively.

**Conclusions:**

We concluded that the high sensitivity of the test permits its use for the identification of patients with GAS. Rapid streptococcal antigen detection tests have been shown to be an important supporting tool in the etiologic diagnosis of pharyngotonsillitis.

## INTRODUCTION

Pharyngotonsillitis (PT) caused by Group A β-hemolytic Streptococcus (SGA) is a common infection in our settings, affecting specially children and young adults. SGA is the main bacterial etiology for acute PT; it affects 15 to 30% of children and teenagers with PT, and 5 to 10% of the adult cases^1^, ^2^, ^3^.

PTs may be caused by viral and bacterial infections; notwithstanding, with rare exceptions (Corynebacterium diphtheriae and Neisseria gonorrhoeae) only SGA caused infections have formal indication of antibiotic treatment^4^, ^5^. In such cases, the antibiotic treatment speeds infection-related symptoms recovery (if used up to 48 hours after symptoms onset), they may help avoid suppurative/non-suppurative complications and community spread^2^, ^6^, ^7^, ^8^, ^9^, ^10^. Rheumatic fever and glomerulonephritis, followed by suppurative complications (abscesses, bacteremia, endocarditis) are the most feared complications^11^.

Since most acute PTs are caused by other agents, viruses for instance, they do not require antibiotic treatment. In this context, it is of the utmost importance that clinicians and otolaryngologists be capable of ruling out streptococcus PT, avoiding inappropriate antibiotic use in the non-streptococcus cases, thus not exposing patients to unnecessary expenses, antibiotic-related risks and increase in bacterial resistance^7^, ^9^.

In the USA, 70% of PTs are treated with antibiotics^4^, ^5^, and it is believed that in Brazil, there may be an even greater number of PTs treated this way.

Due to the variability in strep PT clinical presentations and the large number of other agents capable of producing similar signs and symptoms, it is not always that a clinical diagnosis of SGA causing PT is reliable^10^, ^12^. Thus, physicians may use some laboratorial methods to help attain an etiological diagnosis. During the 80´s, quick tests to detect SGA antigen were introduced in the market, aiming at diagnosing them in minutes. These are easy to handle and interpret methods that may be used in the doctor's office^7^, ^8^. Thus, the quick tests may help in the etiological diagnosis and treatment of streptococcus pharyngotonsillitis. New techniques for the quick detection of SGA antigens have been developed in order to make them more sensitive, easy and inexpensive to use^10^, notwithstanding, its use in public health care facilities, where it is most important, is still scarce because of economic reasons.

The authors aim at investigating both the sensitivity and specificity of the SGA antigen quick detection test, comparing it to culture results in order to define their role in the daily ENT practice

## MATERIALS AND METHODS

**Chart 1:**
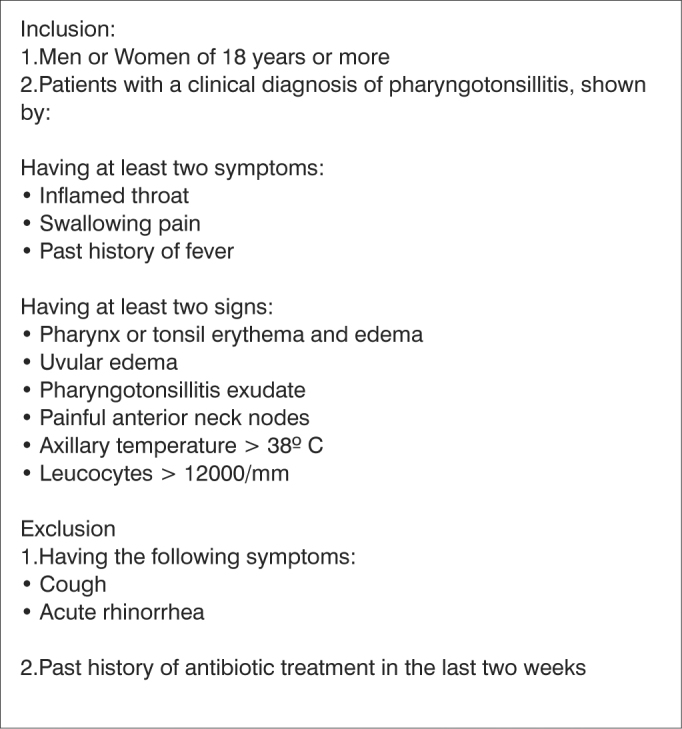
Inclusion and exclusion criteria for the study

81 patients diagnosed with clinical PT, seen at the Otolaryngology ER of the University Hospital of the Medical School of the University of São Paulo, from May 2001 to April, 2002, were selected for the study as long as they fulfilled the inclusion criteria and were not within the exclusion criteria (Chart 1). Fifty five (67%) were men and twenty six were women (33%). Average age was of 39.4 years, varying from 18 to 69 years.

The same team of otolaryngologists took two swabs from all the patients (Culturette; Becton Dickinson, Maryland) simultaneously from the oropharynx posterior wall and from the tonsils. The first of them was taken to the laboratory and put into a 5% goat blood agar dish for 24-48 hours, following conventional biochemistry methods, and the identification was confirmed by the Untek Systems (Biomeniux, USA) test. The other swab was tested in 5 minutes, using the quick test, using the immune-assay optical system, Strep A OIA Max, according to manufacturer's instructions (International Microbio, USA). The lab technician did not have any knowledge about the quick test result. Any streptococcus pyogenes growth in the culture dish was considered a positive culture test.

The quick test results were compared to the culture results and both the sensitivity and specificity of the Strep A OIA Max kit were determined, using the lab culture as the gold standard method.

## RESULTS

We analyzed a total of 81 oropharynx swabs, of which 46 (56%) had positive quick test results and 35 (44%) negative. Thirty three (40.7%) patients had positive streptococcus pyogenes culture (Table 1). There were 2 false-negatives. The quick test sensitivity was of 93.9% (31 of 33), the specificity was 68.7% (33 of 48), the positive predictive value was 67.4% (31 of 46) and the negative predictive value was 94.2% (33 of 35). Of the five positive cultures for other groups of β-hemolytic streptococcus, only one (Group C streptococcus) had a positive quick test.

**Table 1:** Comparison of the quick test with culture in order to detect the SGA antigen in 81 patients with pharyngotonsillitis.

Method	Culture		Total
	Positive	Negative	
IOA quick test			
Positive	31	15	46
Negative	02	33	35
Total	33	48	81

Forty five patients (55.5%) had cultures with other bacteria present.

## DISCUSSION

Like Mayes et al.^7^, we used swabs to collect bacteria samples from the oropharynx of patients, and obtained positive results in almost all the cultures. Of the 81 patients involved in the study, 78 (96.2%) showed bacterial growth in the culture, 40.7% SGA positive, leading us to believe that both the inclusion and exclusion criteria used were adequate and indicated bacterial pharyngotonsillitis, although it may correspond to healthy carrier individuals and/or false positives. In the SGA negative culture patients there was a greater prevalence of saprophyte bacteria in the oropharynx.

We decided to use the blood agar culture medium, in an aerobic environment, because studies that compared different culture mediums showed that this medium was as good as other more selective methods^8^, ^9^, ^13^. The blood agar culture medium is the best test for SGA diagnosis, with a sensitivity between 90 and 95%^6^, ^7^, ^12^. It is very likely that the culture false negative tests are results from patients with a small number of colonies, and many are carriers. However, this test may delay the recognition of a streptococcus-caused PT in 48 to 72 hours, preventing early treatment, and antibiotic therapy would lose its power in alleviating PT symptoms and reduce SGA transmission to other people^14^.

The streptococcus pyogenes prevalence in the references we studied is of about 25% for acute pharyngotonsillitis events^7^, ^8^, ^9^, ^10^. According to Bisno et al., 5% to 10% of adults with acute PT have SGA as the causing agent, however, we found 40.7% among our patients, and this was not surprising for two main reasons: first because the prevalence of this agent in developing countries such as Brazil is markedly higher, due to factors related to precarious basic sanitation and deficient health systems^10^, and also because the inclusion and exclusion criteria were “selective” for SGA.

The criteria were used in an attempt to select streptococci PT, based on the criteria proposed by Centor (pharyngotonsillitis exudate, fever, neck lymph node enlargement and no cough). According to some authors, Centor criteria are more reliable predictive clinical factors for SGA-causing PT diagnosis^4^. There is a 56% positive predictive value when it fulfills all four criteria, especially when applied in high prevalence regions^4^, ^5^, and this was confirmed in our study. However, using only clinical criteria in the diagnosis would still bring about unnecessary treatment for many patients.

The Strep OIA Max kit proved to be practical, easy to use and interpret.

The sensitivity of the SGA antigen quick detection test in the literature varies from 77% to 97%^8^. The great sensitivity variability shown by many authors may be due to some bias, such as the knowledge of the quick test result during the culture colonies count, the culture methods used and the lack of experienced technicians^8^, ^10^. We found high sensitivity (93.9%), however, short of the ideal one proposed by Kellogg and Mozella^13^.

A 68.7% specificity proved to match that of other studies, which reported a variability of 54% to 100%^9^. The increase in the number of patients studied could contribute to improve this parameter. According to Hendley et al., the investigations sponsored by the companies that manufacture the quick test presented better results^8^. Similarly to this author, our study was not sponsored by any company.

Pichichero et al. obtained only 2.4% of false-negatives, suggesting that the quick test could replace culture in the diagnosis of SGA-caused infections^7^. We had two (6%) false-negatives in our study, and this could have happened because of the small quantity of antigens in the oropharynx. Although the test came out negative, we observed bacterial growth in the culture. These cases may be explained by the low colony number, which are only detected in the culture^8^, ^10^. This also happens to SGA bearing individuals, without clinical disease, or when there are antigen blocks by the very anti-streptococcus antigen in the body^13^. Besides, material inadequate collection may also result in false-negatives^8^, ^13^.

False-positives were frequent, and occurred in 15 (32.6%) patients, probably due to the failure in the quick test method^8^, detecting SGA non-specific bacterial antigens, or due to a cross-reaction with other groups of streptococcus, as it happened in one of the cases (Group C streptococcus). The use of mouth washers may also cause false-positives, because they impair the proper growth of organisms in the culture medium^4^, ^8^, however, this possibility was not assessed in our study.

The quick test negative predictive value was of 94.2% and this alerts us for the possibility of 6% of negative tests having positive cultures. Notwithstanding, this represents a small fraction of the population and complications would be very rare, especially among adults - our studied population, where rheumatic fever development is exceptional. This great sensitivity of the quick test could spare patients of being necessarily submitted to culture and being treated with a positive test-based antibiotic therapy. On the other hand, there are authors who advocate culture tests in all negative quick test patients, in order to reduce the risk of complications^7^, ^12^.

The CDC (Center for Disease Control and Prevention) in the USA advises for the treatment of all SGA-caused PT. Cooper et al. suggest that cultures in the USA should not be recommended in the initial assessment of PT patients or to confirm negative quick test results, when these have known sensitivity above 80%^4^. The need to asses this test sensitivity in the Brazilian population and the singular profile of streptococcus PT in Brazil motivated us to carry out this study.

We understand that in negative result quick tests, since the test is very sensitive, we neither order a culture, nor use antibiotic treatment, because it is a very reliable test. In positive tests it is worth to use antibiotic treatment. However, a 67.4% predictive positive test shows that approximately 30% of the cases will be unnecessarily treated. Having seen the high SGA prevalence in our settings, this is a result that is extremely favorable towards the SGA quick test kit in our country. We still lack a cost-benefit study about the use of a quick test in our country. Notwithstanding, in the USA, despite the higher cost of the quick test when compared to culture, there are great savings in the prevention of injudiciously use of antibiotic and additional medical visits for reasons triggered by antibiotic side effects^7^, ^9^.

## FINAL COMMENTS

Streptococcus pyogenes was the first most prevalent organism in bacterial pharyngotonsillitis in our country.

The streptococcus antigen quick detection tests proved to be one important adjuvant weapon in pharyngotonsillitis diagnosis, because it is very sensitive. Thus, negative tests guide diagnosis because of a lack of streptococcus PT and, as such, should be treated only symptomatically, while positive tests guide the diagnosis towards streptococci PT, and should be treated with antibiotics.

We believe that new studies should be made in order to determine the cost-benefit of these detection kits and their impact in the health care economy.
